# Antibiofilm effect of Nano chitosan and calcium hydroxide intracanal medications and their effects on the microhardness and chemical structure of radicular dentine

**DOI:** 10.1186/s12903-025-05462-z

**Published:** 2025-01-20

**Authors:** Maha Nasr, Ahmed Abdou, Dina M. Bassiouny, Reham Hassan

**Affiliations:** 1 Department of Endodontics, Faculty of Oral and Dental Medicine, Egyptian Russian University, Badr City, Egypt; 2 Department of Restorative Dentistry, Faculty of Dentistry, University of Malaya, Kuala Lumpur, Malaysia; 3 Clinical and Chemical Pathology Department, Faculty of Medicine, Cairo University, Cairo, Egypt; 4 Department of Endodontics, Faculty of Dentistry, Minia University, Minia, Egypt

**Keywords:** Antibiofilm, *E. Faecalis*, Fourier transform infrared spectroscopy, Microhardness, Nanochitosan

## Abstract

**Background:**

Disinfection of the root canal system is a challenge to all clinicians, calcium hydroxide Ca(OH)_2,_ one of the most popular intracanal medications used for this purpose, has some unwanted effects on dentine. This study aimed to investigate the antibiofilm effect of Nanochitosan (CSNPs) and Calcium hydroxide Ca(OH)_2_ intra canal medications and their effect on the microhardness and chemical structure of radicular dentine.

**Methodology:**

A total of 52 extracted human mandibular premolars were used. Eighteen premolars were instrumented, sterilized, and inoculated with Enterococcus faecalis *(E. faecalis)* then divided randomly into 2 groups based on the intracanal medication used: group (A) 2% CSNPs gel and group (B) Ca(OH)_2_ paste. Antibiofilm effect was evaluated using bacterial counts. For the effect on microhardness, 56 specimens were subjected to Vicker’s microhardness test before and after 1 week, and 4 weeks of medication application. Twelve cervical radicular dentine specimens (6 samples per group) were examined using Fourier transform infrared spectroscopy and scanning electron microscope.

**Results:**

Both groups showed a significant reduction in the bacterial count at (*p* = 0.008) with no significant difference between them (*P* = *0.605)*. Ca(OH)_2_ showed the highest reduction in the microhardness compared to CSNPs groups after 1 week (*P* = *0.0495)* and after 4 weeks (*P* = *0.0495)*. FTIR spectrum results revealed that the control group (no treatment) showed the highest significant Phosphate: amide ratio compared to both CSNPs and Ca(OH)_2_ at *p* = 0.006. SEM images revealed absence of discernible smear layer in CSNPs treated samples after 4 weeks and all the dentinal tubules were open.

**Conclusions:**

Nanochitosan gel could be considered as a viable option as an intra canal medication.

## Background

Success of root canal therapy relies on complete pulp space debridement by elimination of pulpal tissues, dentine debris, and microorganisms [1]. Complete eradication of intra-radicular infections with mechanical instrumentation alone is impossible due to the complexity of the root canal system [2]. Therefore, chemical, and mechanical preparations must be done in conjunction with each other to obtain such promising results [3]. Moreover, in order to allow for healing, any antigen that may be left in the canal after elimination of the microorganisms must be neutralized. Therefore, the combined chemo‑mechanical treatment should be followed by a suitable intracanal medicament in infected canals [4].

Currently used intracanal medicaments are phenolic compounds such as camphorated monochophenols, gluteraldehyde, calcium hydroxide, and some antibiotic pastes. These compounds despite having good antibacterial effects, have detrimental effects on the dentine matrix either due to the acidity of the medication as in case of antibiotic pastes [5], or the very high alkalinity of calcium hydroxide, which was proved to reduce the microhardness of root dentine by a disruption of the link between the hydroxyapatite crystals and the collagenous network in dentine [6].

Despite its rather detrimental effects on dentine matrix and its inability to completely eradicate some resistant strains of *E. faecalis*, Calcium Hydroxide (Ca(OH)_2_) is the most effective intra canal medicament currently used [7]. However, possible side effects, safety concerns, and sometimes the ineffectiveness of conventional intracanal medications, natural and more advanced preparations are being constantly developed and tested [8]. Modern advances in nanotechnology provide novel and promising opportunities to eradicate bacteria, disrupt biofilm and control dentine infection in root canal therapy [9].

Chitosan (CS), a natural polysaccharide obtained from the shells of crustaceans, is nontoxic, biocompatible, and biodegradable. It’s been introduced in the field of endodontics as a broad-spectrum antimicrobial agent with significant chelating effects [10]. Nanoparticles, having polycationic/polyanionic nature, great surface area and charge density, are expected to provide better interaction with the bacterial cells [11]. Therefore, chitosan nanoparticles (CSNPs) were engaged in different fields of healthcare including endodontics because of their potential antibacterial activity [9, 12] .

However, to our knowledge, the impact of CSNPs application as intracanal medication on both the microhardness of the root dentine and the chemical changes in the dentine structure has not yet been studied. Thus the aim of this study was to evaluate the antibiofilm effects of CSNPs and Ca(OH)_2_ intracanal medications against mature *E-faecalis* biofilms in the root canals of extracted mandibular premolars and to investigate their effects on the microhardness of the root dentine and the structure of the dentine surface. The null hypothesis tested is that no significant differences would be found between the tested groups.

## Materials and methods

**Fig. 1:**
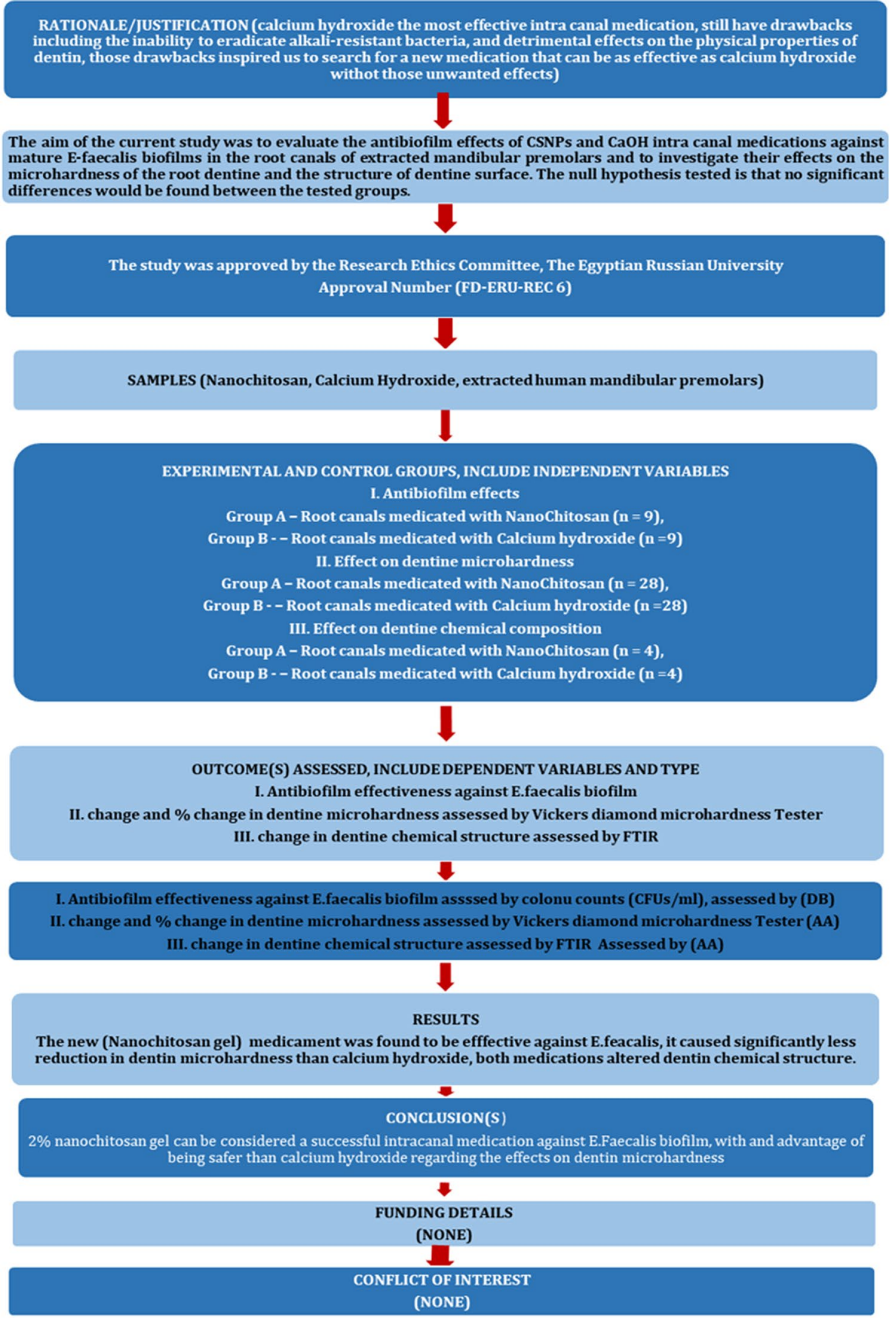
PRILE 2021 flowchart

The manuscript of this laboratory study has been written according to Preferred Reporting Items for Laboratory studies in Endodontology (PRILE) 2021 guidelines (Nagendrababu et al., 2021; Fig. 1).

### Preparation of CSNPs

CSNPs were prepared according to the ionotropic gelation process [13]. In brief, CS (0.5%) in acetic acid (1%) was dissolved distinctly in distilled water in room temperature using magnetic stirring. The poly anion sodium tripolyphosphate (TTP), used as a cross-linking agent, was also dissolved separately at 2 mg/ ml. TTP solution was added in drops into the CS solution using a 1mL syringe, and the pH was adjusted at 4. Based on the CS concentration being used, TPP solutions yielded a final CS to TPP mass ratio of 3:1. Blank nanoparticles were obtained upon the addition of a TPP aqueous solution to a CS solution.

### CSNPs characterization

**Fig. 2:**
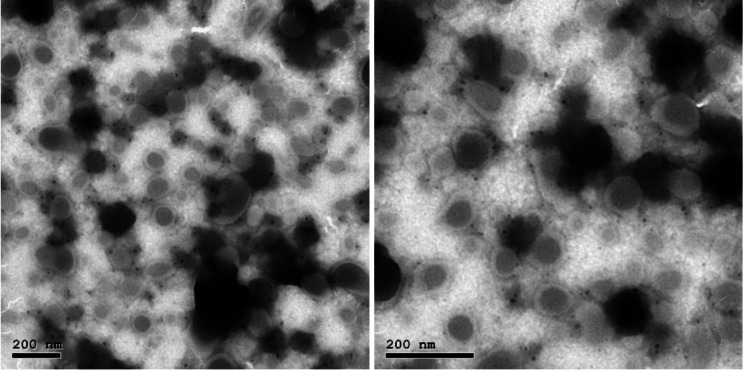
TEM images taken for the CSNPs nanoparticles at magnification scale 200 nm

For further assurance, the size and morphology of the CSNPs nanoparticles were observed under transmission electron microscope (TEM: JEM-2100, JEOL Ltd, Japan) performed on JEOL JEM-2100 high resolution at an accelerating voltage of 200 k. TEM revealed the formation of uniform spherical shaped particles, with size distribution less than 50 nm (Fig. 2).

### CSNPs gel

The prepared CSNPs were redispersed in distilled water to get 5 ml, 2%w/v with stirring for 30 min. 0.25gm of Hydroxypropyl methyl cellulose (HPMC; Loba CHIME, India) was sprinkled gently and gradually over the solution under mild temperature 35 °C with vigorous stirring to get homogenous gel.

### Sample selection and preparation

**Fig. 3:**
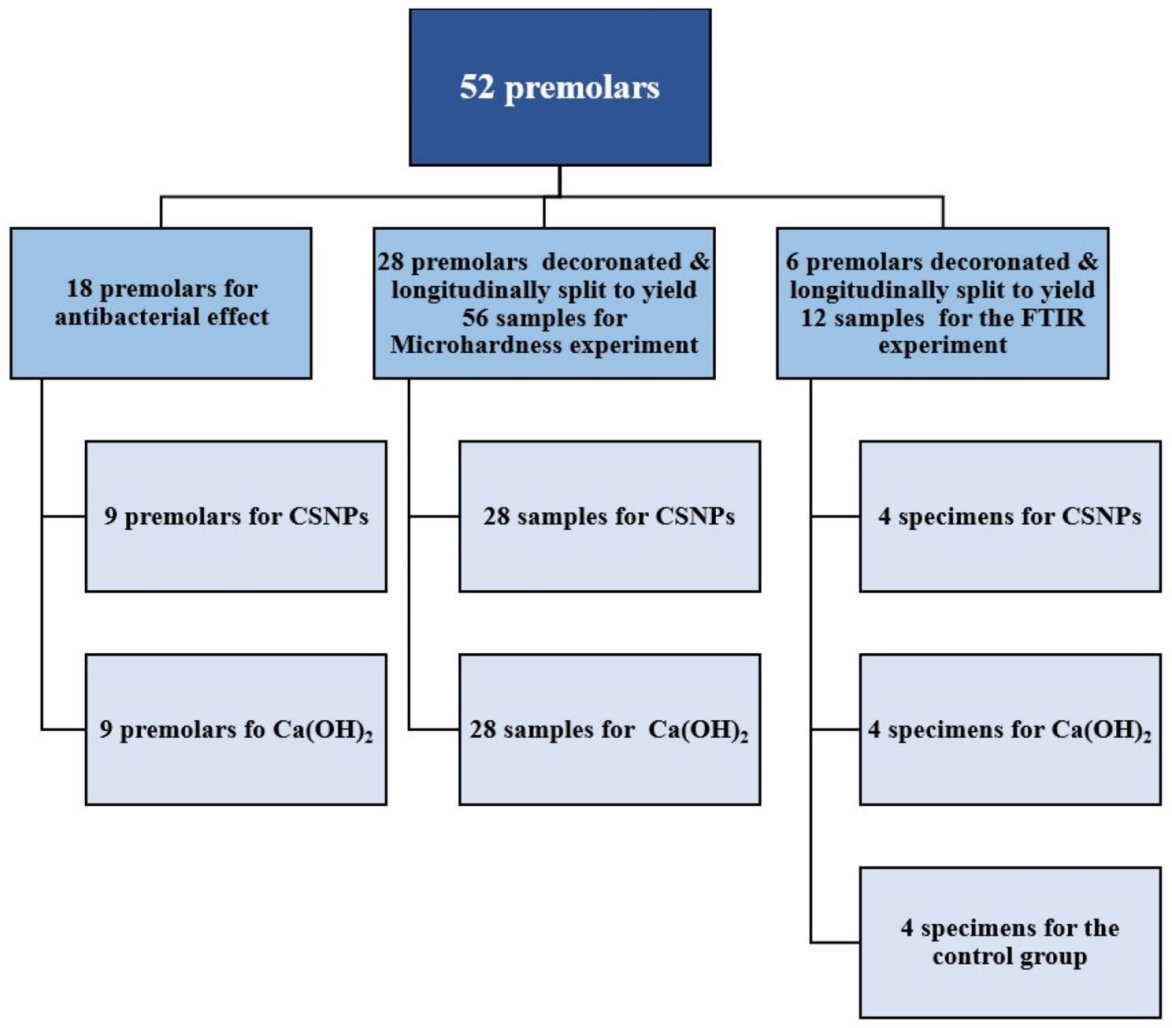
Samples classification

After the approval of the study protocol by the faculty local research ethical committee (no FD-ERU-REC 6), fifty-two human extracted mandibular premolars with mature apices extracted for orthodontic reasons were collected from the university’s department of Oral Surgery (Fig. 3). Teeth were placed in 5.25% of sodium hypochlorite (NaOCl; CHLORAXID 5,25% EXTRA, Cerkamed, Pawłowski, Poland) for 15 min for disinfection and removal of residual tissues, scaled using ultrasonic scaler to remove any surface debris, and examined under a surgical operating microscope (Zumax, Zumax Medical Co., Ltd., Jiangsu, China) under 12x magnification. Teeth with external defects, caries, fractures, cracks, incompletely formed apices or resorptions were excluded and replaced. Preoperative radiographs from both the buccolingual and mesiodistal directions were taken to demonstrate the presence of a single canal. The inclusion criteria included complete root formation, no internal root calcification, no internal or external root resorption. Teeth with root curvature 0°–10° measured using Schneider method [14] were selected and involved in the study.

## Assessment of antibacterial activity

### Sample size calculation

By adopting an alpha (α) level of 0.05 (5%), a beta (β) level of 0.05 (5%) i.e., power = 95%, and an effect size (f) of (2.115747) calculated based on the results of Degaldo et al. [15]; the predicted sample size was a total of 16 samples. Sample size calculation was performed using G*Power version 3.1.9.7. Sample size was increased to a total of 18 samples to account for any sample loss.

### Sample preparation

Eighteen intact premolars were selected, access cavities were prepared using #2 round burs and tapered stones with rounded ends. Working length was then obtained by subtracting 1 mm from the tooth length determined by extruding a #15 K-file (Dentsply/Maillefer, Ballaigues, Switzerland) beyond the apex followed by its withdrawal to be coincident with the apex. Mechanical preparation was done using rotary ProTaper Universal (Dentsply/Maillefer, Ballaigues, Switzerland) files up to size F4 connected to an endodontic motor (Endo-Mate TC2; NSK Nakanishi, Tochigi, Japan) at speed of 300 RPM and 2 Ncm torque, according to the manufacturer’s instructions. During instrumentation, canals were irrigated using 5 ml 2.5% sodium hypochlorite using a 30-gauge needle (Endo Top irrigation needles, Cerkamed, Pawłowski, Poland) adapted to a disposable plastic syringe AND positioned 2 mm short of the working length. Smear layer was removed using 5 ml of 2.5% NaOCl (Cerkamed, Pawłowski, Poland) for 1 min and 5 ml 17% EDTA (Cerkamed, Pawłowski, Poland) for 1 min. After chemo-mechanical preparation, specimens were autoclaved to ensure sterility before introducing the bacteria, then all roots were coated with a double layer of colorless nail polish and apices were sealed with resin composite restorations [16].

### Bacterial growth

*E. faecalis* (American Type Culture Collection 29212), was obtained and activated in the Laboratory of Microbiology. The bacteria were cultivated in brain heart infusion (BHI) broth for 18–24 h at 37 C in an incubator.

### Experimental root canal infection

In each of the 18 previously sterilized samples, 1 mL *E. faecalis* (6.3 × 108 colony-forming units (CFUs) culture broth was inoculated into the root canal. All specimens were incubated aerobically at 37 °C. The *E. faecalis* culture was maintained for 21 days to promote bacterial growth, with the BHI being renewed every 72 h. All procedures were performed under aseptic conditions in a laminar flow hood [17].

### Pre-treatment culture and analysis

After this period, each tooth was irrigated with sterile saline, absorbent paper points were inserted in each tooth and transferred to individualized phosphate buffered saline (PBS) solution tubes. Each tube was cultured using 10-micron sterile culture loops on bile esculin and CLED agar plates to get the colony count (CFUs/ml) of inoculated and incubated Enterococcus spp [17]. Confirmation of culture yield was done using gram stain, catalase test and Bile esculin subculture [18, 19].

### Classification of samples and medicament application

After the period of incubation and pre-treatment culture, the 18 samples were divided equally and randomly using a random group allocation online software (https://www.ramdomizer.org) into two groups according to the medicament used:

Group A: 2% Nano chitosan (CSNPs) gel was used as an intracanal medication.

Group B: Calcium hydroxide (Ca(OH)_2_) was used as an intracanal medication.

Each intracanal medication was applied under sterile conditions. After the excess material was removed, access cavities were sealed with temporary restorative filling material (Cavit, 3 M ESPE). Both groups were incubated for 1 week at 37 °C in an incubator.

### Microbiological analysis

After the respective treatments and the incubation period, each specimen was irrigated with 10 ml of sterile saline to flush away any medications. Then a sterile absorbent paper point (Dentsply Sirona, York, Pennsylvania) was inserted into each root canal space and agitated in a circumferential way with intentional touching of the walls for 30 s. Absorbent paper points were transferred to a tube containing 1 mL sterile phosphate buffered saline (PBS). The material was homogenized and cultivated on the surface of the bile esculin and CLED agar in plates and incubated for 24 h at 37 °C. After the incubation period, CFUs were counted manually on the plates [17]. 

## Microhardness experiment

### Sample size calculation

According to the study by P. Serdar Emyirili et al. [20] the mean (SD) percentage reduction of microhardness of roots treated with calcium hydroxide for one month were found to be 45.3 (10.64). The sample size for the current study was calculated using an estimated Cohen’s d effect size of 0.75, a type I error of 0.05 and a power of 0.8. Twenty-eight samples per group were required to detect a significant difference between the two groups regarding microhardness after 1 month. The Sample size was calculated using the G Power software version 3.1.9.7.

Intact teeth were decoronated using diamond coated discs and a 5-mm cervical cylinder was prepared from each root before application of the intra canal medications. Each root cylinder was sectioned longitudinally using a water – cooled safe sided diamond disc and then sectioned with a spatula into two halves; thus, each tooth yielded two samples which were coded as A and B, so that each tooth was allocated in both groups.

### Sample classification

56 samples (from twenty-eight teeth) were divided randomly into two groups (*n* = 28) according to the medicament used:

Group A: 2% CSNPs gel was used as an intracanal medication.

Group B: Ca(OH)_2_ was used as an intracanal medication.

In each group the effect of the intracanal medication on dentine microhardness was measured at three time intervals; before (baseline), 1 week and 4 weeks after placement of the medication in the root canal.

All samples were rinsed thoroughly with saline and mounted on acrylic cylinders using self-cure acrylic resin with the intracanal-medicated surface facing upwards. The surface of the specimens was finished using silicon carbide paper with 400, 800 and 1200 grit and a polishing cloth with a diamond suspension (1 mm; Struers). Pre-treatment microhardness measurements were performed using a Vickers diamond microhardness Tester (Nexus 4000/60, Innovatest, Maastricht, Netherlands) on the coronal side of each root cylinder. The microhardness measurements were taken at three different points at a depth of ~ 1 mm from the pulp-dentine interface (using the digital caliper attached to the microhardness tester). Each measurement was carried out using a 50 g (HV 0.05) load for 10 s dwell time. The representative microhardness values were reported as the mean of the three indentations.

### Intracanal medicament application and final measurement

The intracanal medication was tamped into the canal space, ensuring that the pulpal surface of each specimen was fully covered. Specimens were then stored for either one or four weeks at 37 °C at 100% relative humidity. Each specimen was hydrated with 0.05 mL of deionized water weekly. Each root canal was then thoroughly irrigated with distilled water for 30 s to remove the medication, and post-treatment microhardness measurements were taken as described previously. The percentage change in microhardness for each sample was calculated as follows:

(Post-treatment microhardness − pre-treatment microhardness) × 100 / pre-treatment microhardness.

## Fourier transform infrared spectroscopy (FTIR) experiment

### Sample size calculation

By adopting an alpha (α) level of 0.05 (5%), a beta (β) level of 0.05 (5%) i.e., power = 95%, and an effect size (f) of (1.5) calculated based on the results of Yassen et al. [21] the predicted sample size was a total of 12 samples for 3 different groups. Sample size calculation was performed using G*Power version 3.1.9. 7.

### Sample preparation

Six intact mature single-rooted premolars were selected according to the same criteria described previously. A cervical 4-mm root cylinder from each tooth was obtained and sectioned longitudinally yielding 12 samples, each sample was ground flat to a uniform thickness with 1,200 grit silicon carbide grinding paper (Buehler Ltd., Lake Bluff, IL, USA) under constant water-cooling. To remove the smear layer, specimens were ultrasonicated for 5 min under distilled water. Samples were randomly assigned to the three treatment groups (*n* = 4 per group) as follows, group (A): control group where no medications were added, group (B): CSNPs group and group (C): Ca(OH)_2_ group.

### Medicament application and FTIR measurement

Each dentine specimen was placed in a 2-mL conical sample cup (Fisher Scientific, Florence, KY, USA) containing 0.15 mL of one of the treatment pastes which was sufficient to completely cover the pulpal surface of each sample. The sample cups were then stored for four weeks at temperature of 37 °C with 100% relative humidity. After four weeks, the specimens were thoroughly rinsed with distilled water to remove any visible paste and then subjected to 15 min of ultrasonication under distelled water before being air-dried.

The chemical structure of untreated (control) and treated dentine specimens was analyzed using a 4100 FTIR spectrophotometer (Bruker Optik GmbH) with a diamond ATR setup. The dentine specimens were placed on a standard FTIR sample holder with a 2.5-mm diameter opening, and three spectra were obtained from each specimen between 400 and 4000 cm-1 at a resolution of 1 cm-1 using 100 scans. Each obtained spectrum was then processed by smoothing, baseline correction, and normalization against the amide I peak using a specific Spectra Manager CFR software (JASCO Inc.). The effect of the two medicaments on collagen and apatite composition of the dentine surface was assessed by determining the mineral matrix ratio (ratio of integrated areas of phosphate v1 and v3 peaks to the amide I peak) [22]. The final ratio assigned for each dentine specimen represented the average of the ratios obtained from the three spectra.

### Scanning electron microscopy (SEM)

SEM analysis aimed at detecting any morphological changes of root canal dentine. Two samples were randomly selected from each group that had undergone the FTIR experiment. Each half of the root cylinder was irrigated with 5 mL of deionized water, followed by sonication in deionized water for 5 min, and then drying for 48 h. Specimens were coated with gold/ palladium for 70 s using a sputter coater (Polaron, Agawam, MA, USA) and images were taken from the treated root canal surface area of the specimens with a JEOL 7800 F scanning electron microscope (JEOL, Peabody, MA, USA) in secondary electron imaging mode.

### Statistical analysis

Data was explored for normality using Shapiro Wilk test. Mann Whiteny U test was used to compare between tested groups while Wilcoxon signed rank test was used to compare between follow-up periods for microhardness and Log10/CFU. The phosphate: amide ratio showed normal distribution, so one-way ANOAVA was used to compare between tested groups followed by Tukey’s test for pairwise comparison. A significant level was set at 0.05. (IBM SPSS, Armonk, NY, USA).

## Results

Regarding antibacterial efficacy (Table 1), both groups showed a significant reduction in the bacterial count at *P* = 0.008. with no significant difference between them (*P* = 0.605). Both groups significantly reduced dentin microhardness (Table 2). Ca(OH)_2_ showed the highest reduction on the microhardness compared to CSNPs groups after 1 week (*P* = *0.0495)* and after 4 weeks (*P* = *0.0495)*. Four Weeks storage resulted in significant reduction in microhardness compared to 1 week storage for both materials (*P* < *0.05)*.

**Table 1:** Bacterial count (log 10 CFU) for tested groups

		CSNPs				Ca(OH)2				*p*-value
		Mean	SD	Min	Max	Mean	SD	Min	Max	
Log10/CFU	Preoperative	4.8	0.1	4.7	4.9	4.7	0.2	4.3	4.9	0.222
	Postoperative	1.0	1.3	0.0	2.8	1.3	1.5	0.0	3.3	0.605
p-value		0.008				0.008				

**Table 2:** Percent of change in hardness after 1 week and 1 month of the use of the intracanal medication

		CSNPs				Ca(OH)2				*p*-value
		Mean	SD	Min	Max	Mean	SD	Min	Max	
% of change	1 week	-2.2	1.3	-3.0	-0.8	-9.1	2.6	-12.0	-7.0	0.0495
	4 weeks	-10.2	1.6	-11.5	-8.4	-17.2	3.6	-21.4	-14.7	0.0495
p-value		0.0487				0.040				

**Fig. 4:**
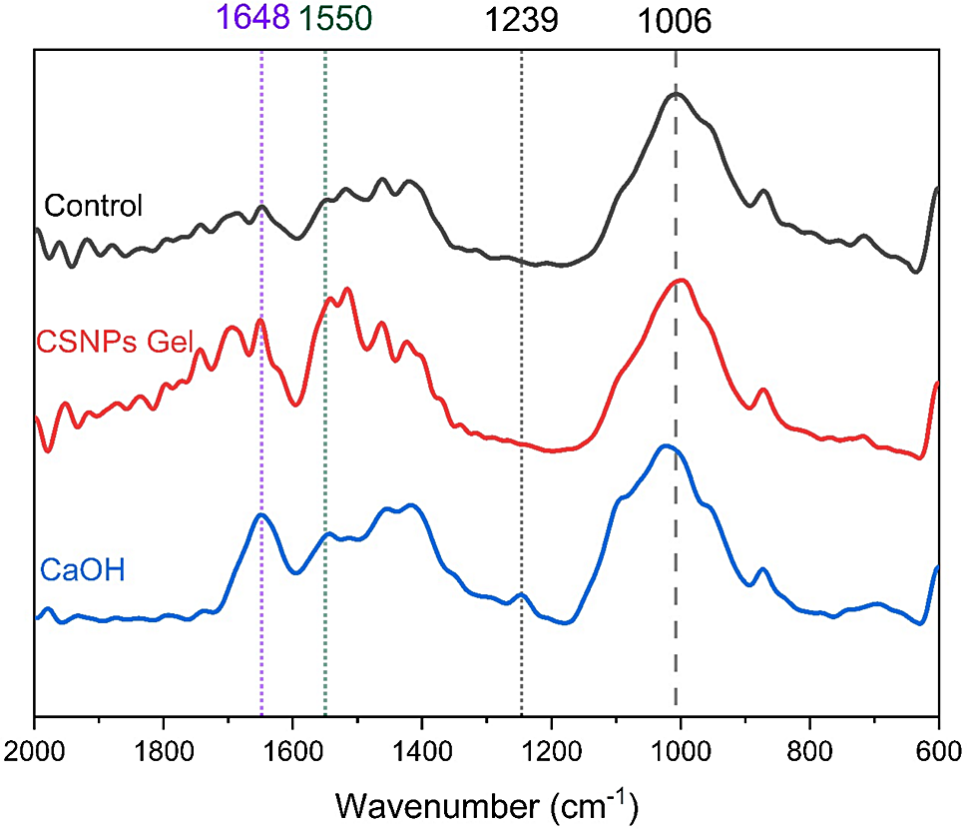
FTIR Spectrum of dentin treated surfaces. The peak value at 1648 cm^− 1^ is the characteristic peak of amid I band, and 1550 cm-1 of amide II band, while the 1245 cm-1 indicated for amide III band. The peak at 1006 cm-1 indicates PO4^3−^ V_3_

Results of Phosphate: amide ratio is presented in (Table 3) and representative FTIR spectrum is presented in (Fig. 4). The control group (no treatment) showed the highest significant Phosphate: amide ratio compared to both CSNPs and Ca(OH)_2_ at *P* = 0.006. There was no significant difference in the Phosphate: amide ratio between CSNPs and Ca(OH)_2_. FTIR spectrum showed the presence of peak value which is the characteristic peak of amid I band at 1648 cm^− 1^, and amide II band 1550 cm^− 1^ for all groups while the amide III band at 1245 cm^− 1^ was profound for Ca(OH)_2_ treated dentin.

**Table 3:** Phosphate: amide ratio for tested groups

	Control				Ca(OH)2				Nano chitosan				*p*-value
	Mean	SD	Min	Max	Mean	SD	Min	Max	Mean	SD	Min	Max	
*Phosphate: amide %*	6.25^a^	0.87	5.4	7.8	3.10^b^	1.61	1.3	5.2	3.62^b^	1.9	0.4	5.9	0.006

### Different letter within row indicates significant difference ta *p* < 0.05 (Tukey’s HSD adjusted)

**Fig. 5:**
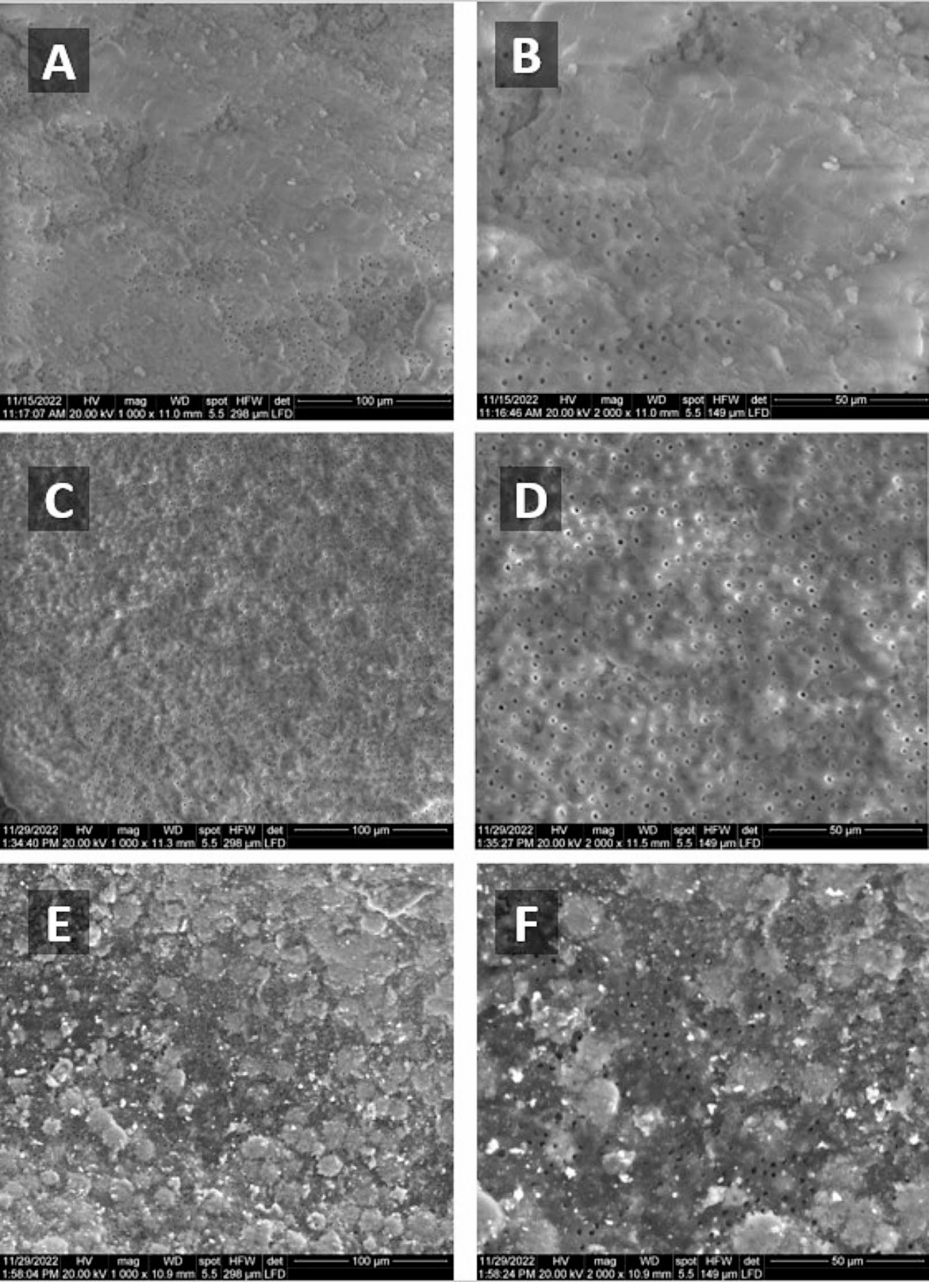
SEM images for radicular dentine surfaces. (**a**-**b**) Baseline group showed the presence of a smear layer in root canals of untreated control dentine. (**c**-**d**) CSNPs group shows no visible smear layer with opening of all dentinal tubules after intracanal medication application for 4 weeks. (**e**-**f**) Ca(OH)2 group: presence of smear layer and remnants of the Ca(OH)2 covering the surface of dentine

The SEM images of radicular dentine surfaces were examined in different groups with the aim of evaluating the presence of the smear layer after canal treatment (Fig. 5). The control group showed the presence of a smear layer in root canals of untreated dentine, depicted in images a, b. Nevertheless, following intracanal medication using the CSNPs, as shown in images c, d, no discernible smear layer could be observed after 4 weeks, and all the dentinal tubules were opened. On the other hand, images e, f showed the presence of a smear layer in Ca(OH)_2_ group and remnants of the Ca(OH)_2_ were present covering the surface of dentine.

## Discussion

The anatomical complexity of different root canal systems renders the use of potent antimicrobial chemicals a must for successful root canal treatment [23]. However, almost all the chemicals used during the process of root canal treatment may induce physical and chemical changes in the root dentin and can affect its mechanical properties [24]. The application of chitosan in different fields is ecologically interesting, because it is the most prevalent element in nature after cellulose [25]. The clinical use of new formulations must be preceded by laboratory studies that thoroughly investigate their benefits and consequences. Although previous attempts have been made to assess the antibacterial efficacy of chitosan nanoparticles [16, 26], their impact on the microhardness and structural integrity of dentin remains unexplored. This study aimed to fill this gap by investigating antibiofilm effects of CSNPs and Ca(OH)_2_ intra canal medications against mature E-faecalis biofilms in the root canals of extracted mandibular premolars and to investigate their effects on the microhardness of the root dentine and the structure of dentine surface, thereby contributing crucial insights into their broader applications in the field of endodontics.

*E. faecalis* was identified as an important microorganism using culture-dependent and molecular techniques [27]. It was detected as the most prevalent microorganisms in root canal treated teeth (36.6%) using culture methods [28] and at a prevalence of up to 77% of failed endodontically treated teeth using molecular methods [29]. Even though *E. faecalis* was not the most abundant bacteria found, it was detected at higher quantities in secondary apical periodontitis compared to primary infection. Consequently, maintaining these microbes in the root canals can pose difficulties for the success of endodontic therapy, and the presence of a potent antimicrobial agent for *E. faecalis* in root canals is crucial [28, 29]. 

Calcium hydroxide the most commonly used intracanal medication is known for its alkalinity and its ability to destroy bacterial DNA [30]. However, dentine exerts a buffering action that can neutralize the action of Ca(OH)_2_ [31] giving chance to many bacterial species especially *E. Faecalis* that is capable of resisting high pH up to 9 or 10 to survive treatment in the infected root canals [27]. The inability of Ca(OH)_2_ to totally eradicate microbial biofilms together with its untoward effects on dentine structure especially after long term application have provoked researchers to look for natural alternatives [16].

CSNPs with an average size of up to 97 nm were demonstrated to have a strong bactericidal effect against both Gram-positive and Gram-negative bacteria [32]. Therefore, in the current study, 50 ± 5 nm CSNPs were used. As an irrigating solution, CSNPs solution demonstrated a bactericidal effect against *E. faecalis* similar to that of 2.5% Sodium hypochlorite [33]. It was also reported to be significantly more effective against the microbiota of primary endodontic infections than either 5.25% sodium hypochlorite or 2% Chlorohexidine [12]. Shrestha et al. 2010, on the other hand have demonstrated that 2% CSNPs solution had significant antibiofilm effects against *E. faecalis* biofilm, However, it could not totally eradicate it even after 72 h, this was attributed to the presence of a barrier-acting extracellular polymeric matrix in the biofilm that hinders the CSNPs penetration, making a longer contact duration necessary [34]. That is why in the current study 2% CSNPs were used in a gel form as an intracanal medicament to be able to sustain the antibiofilm effects for a sufficient duration. Thus, the current study aimed at exploring the antimicrobial efficacy of 2% CSNPs as an intracanal medication against *E. faecalis* biofilm versus that of Ca(OH)_2_ and their possible effects on the microhardness and surface structure of root canal dentin.

The bacterial culture method was used in the current study to assess the antibacterial effects since it is broadly available, simple, and reliable thus permits accurate quantification of viable cultivable organisms in samples [35].

2% CSNPs gel significantly reduced the number of colony forming units per milliliter (CFU/ml) of *E. faecalis* (*P* = 008). Although, CSNPs group showed a lower mean log/10 CFUs (1 ± 1.3) compared to the Ca(OH)_2_ group (1.3 ± 1.5), the difference was not statistically significant (*P* = 0.605), this finding came in agreement with the findings of Hussein et al. (2019) who compared CSNPs to Ca(OH)_2_ and Di-antibiotic paste and found that all three medications could eradicate *E. faecalis* biofilm with no significant difference between them. On the other hand, our finding contradicts the findings of Sireesha et al. (2017) who found CSNPs to be significantly more effective than Ca(OH)2 against *E. faecalis*. This difference in findings could be attributed to the different methodologies used where they tested CSNPs against *E. faecalis* suspension rather than biofilm, and evaluated the antibacterial effects of the tested medicaments by measuring inhibition zones.

To reduce the impact of the structural differences between different teeth and to establish a reasonable assessment for the impact of the chelating agents on dentine surface, microhardness measurements were done for each tooth at baseline (untreated) and posttreatment for both groups (one tooth half per group, Fig. 3).

The root canals were left mechanically unprepared, to avoid the formation of smear layer. This phase was intentionally left out to avoid any potential alterations in the measurements bought on by ions integrated into the loosely attached smear layer [37].

Intracanal medicaments are intended to remain in the canal for extended periods depending on the clinical situation [38]. The durations evaluated here represent the typical period that intracanal medications are used in various clinical scenarios. As per the guidelines of the International Association of Dental Traumatology (IADT), non-setting Ca(OH)_2_ should be used for up to 1 month prior to the final obturation of the root canal system for avulsed teeth management [39].

In the current study, both materials used as intracanal medication for endodontic disinfection caused significant reductions in microhardness at both time intervals. However, calcium hydroxide showed the highest reduction in microhardness of 9.1% compared to CSNPs gel 2.2% after 1 week (*P* = 0.0495). Moreover, both groups showed a significant reduction in the microhardness over a period of one month when compared to one week (*p* < 0.05). At both time intervals, the experimental groups showed significantly less microhardness compared to the baseline. The mean percentage of reduction of the microhardness after 1 month in the Ca(OH)_2_ group 17.2% was also significantly higher than the CSNPs group 10.2% (*P* = 0.0495).

Studies have shown that long-term intracanal dressing with calcium hydroxide alters the physical characteristics of dentine. This might be explained by the modifications made to the organic matrix as a result of the high alkalinity of calcium hydroxide. Rosenberg et al. (2007) observed that Ca(OH)_2_ can dissolve, denature, or neutralize acidic organic components like phosphate and carboxylate groups in dentine, thus disrupting the bond between collagen fibrils and hydroxyapatite crystals. As a result, the tooth structure becomes weaker, which increases the risk of root fracture and speeds up the propagation of fatigue cracks under cyclic stresses [38].

Chitosan is well known for having a strong capacity to chelate various metallic ions [40]. Two theories currently attempt to explain the chelation process of chitosan. According to the bridge model [41], two or more amino groups from a chitosan chain attach to the same metal ion. According to the second hypothesis, just one of the substance’s amino groups is engaged in the binding process because the metal ion is “anchored” to the amino group [42]. A chain made up of several chitin dimers forms the chitosan polymer. The chitin dimer displays two nitrogen atoms with pairs of free electrons, which are similar to those in the EDTA molecule and are in charge of the ionic interaction between the metal and the chelating agent. The amino groups in the bipolymer are protonated in an acidic environment, producing an overall position charge (-NH3+). For adsorption to take place, this form must be attracted to other molecules [43]. The mechanisms of adsorption, ion exchange, and chelation are most likely responsible for the complexes that develop between chitosan and metal ions.

Machado-Silveiro et al. 2004 [44] concluded that 0.1, 0.2 and 0.37% solutions of chitosan, used for 5 min, promoted exaggerated expansion of the tubular diameter, degrading dentin as the concentration increased. It has been demonstrated that the chelating effect increases with the solution concentration. The concentration of 2% chitosan was ten times that of 0.2 concentration, which probably intensified the demineralizing action of this solution [45]. This could explain the reduction of microhardness caused by the CSNPs intracanal medication used in the current study.

ATR-FTIR was used to examine the changes in chemical integrity of superficial radicular dentine following the application of CSNPs and Ca(OH)_2_ in an attempt to better understand the effect of intracanal medications on radicular dentine. In all dentine slices, the hydroxyapatite crystals, and the visible wave number was 1006 cm^− 1^ and the anti-symmetrical stretching peak of P–O bond was located at the peak of PO4^3−^. The peaks for amide I and II were visible for all groups. However, an increase in the peak intensity was observed for both Ca(OH)_2_ and CSNPs. Interestingly, the amide III at 1245 cm^− 1^ was showing clearly for Ca(OH)_2_ treated dentine. Which might indicate a higher demineralization effect of Ca(OH)_2_ compared to the CSNPs group which might validate the significantly higher reduction in microhardness of roots treated with Ca(OH)_2_. Moreover, the significant reduction in phosphate/amide ratio in pretreated dentin in both groups indicates the formation of collagen-rich matrix on the surface and slight demineralization of the surface of the radicular dentine, which was confirmed by the SEM images.

Despite the promising results obtained from this study, one of the limitations that must be taken into consideration is that the efficacy of CSNPs intracanal medication against the diverse microbiota of endodontic infections need to be thoroughly investigated before evaluating this approach in clinical trials. The antibacterial effects of CSNPs depend on several factors, including and not limited to concentration, where, increasing concentration of the tested chitosan-based materials, decreased microbial survival in a dose-dependent manner [13]. Incorporation of trace amounts of other elements such as silver nanoparticles as well as using CSNPs combinations with other intra-canal medications such as calcium hydroxide and chlorhexidine gel can enhance the antimicrobial effects of CSNPs [13, 16, 46]. Other limitations of this study include, testing the microhardness of dentine in the cervical region of the root only and also not measuring the calcium ions level in the FTIR. Further studies evaluating the interaction between root canal filling materials and CSNPs treated dentine are needed. Additionally, in vivo clinical studies are required to evaluate the long-term survival of teeth treated with CSNPs. In conclusion, the findings of this study provide a basis for further research on the potential clinical application of CSNPs as an intracanal medication for endodontic treatment.

## Conclusions

2% Nanochitosan intracanal medication exhibited a potent effectiveness against *E. Faecalis* biofilm compared to Calcium hydroxide, though the difference was not significant. CNPs had the advantage of a significantly lower reduction in dentine microhardness over calcium hydroxide after 1 and 4 weeks of application. Both medications altered the chemical composition of radicular dentin surface. Nanochitosan intracanal medication could be considered as a viable option as an intra canal medication, it is also deemed safer than calcium hydroxide for long-term application in terms of dentine microhardness and integrity.

## Data Availability

Data is provided within the manuscript or supplementary information files. The data that support the findings of this study are available in request from the corresponding author.
